# Supplementary material for: Associations of body adiposity index, body mass index, waist circumference, and percentage of body fat in young female Emirati adults
Source: PLoS One. 2024 May 6;19(5):e0302779. doi: 10.1371/journal.pone.0302779 (PMC11073684; doi:10.1371/journal.pone.0302779)
Supplement: Attachment — Submitted filename: Response 8.11.23.docx [file pone.0302779.s002.docx]

8^th^ November 2023

Dear Editorial Board,

**Reference: PONE-D-23-35965**

Title: Associations of body adiposity index, body mass index, waist circumference, and percentage of body fat in young female Emirati adults

**Comment**: please report in the Methods section the dates when data were accessed for research purposes and questionnaire based study was conducted.

**Response**: We have addressed the above comment and have revised the Methods Section in our manuscript accordingly. We have uploaded both a marked-up and clean version of our manuscript.

Kind regards,

Dr. Dalia Haroun

Associate Professor

Zayed University-Dubai-United Arab Emirates

Email: [dalia.haroun@zu.ac.ae](mailto:dalia.haroun@zu.ac.ae) Tel: +971-507135613
