# Supplementary material for: Associations of body adiposity index, body mass index, waist circumference, and percentage of body fat in young female Emirati adults
Source: PLoS One. 2024 May 6;19(5):e0302779. doi: 10.1371/journal.pone.0302779 (PMC11073684; doi:10.1371/journal.pone.0302779)
Supplement: Attachment — Submitted filename: Response to Reviewers.docx [file pone.0302779.s003.docx]

***Editor Comments***

**1. Please ensure that your manuscript meets PLOS ONE's style requirements, including those for file naming. The PLOS ONE style templates can be found at** 
[**https://journals.plos.org/plosone/s/file?id=wjVg/PLOSOne_formatting_sample_main_body.pdf**](https://journals.plos.org/plosone/s/file?id=wjVg/PLOSOne_formatting_sample_main_body.pdf) **and** 
[**https://journals.plos.org/plosone/s/file?id=ba62/PLOSOne_formatting_sample_title_authors_affiliations.pdf**](https://journals.plos.org/plosone/s/file?id=ba62/PLOSOne_formatting_sample_title_authors_affiliations.pdf)

Response: We have checked that the manuscript meets PLOS one’s style requirements.

**2. Please provide additional details regarding participant consent. In the ethics statement in the Methods and online submission information, please ensure that you have specified (1) whether consent was informed and (2) what type you obtained (for instance, written or verbal, and if verbal, how it was documented and witnessed). If your study included minors, state whether you obtained consent from parents or guardians. If the need for consent was waived by the ethics committee, please include this information**.

Response: informed written consent was collected from all participants included in the study.

This has been highlighted in the Material and Methods section.

***Reviewer Comments***

**Reviewer #1:** While validating the use of BAI in estimating % body fat among Emirati women is novel, given the small sample size and heterogeneity of the population, concluding BMI is superior to BAI in predicting %BF might not be scientifically sound. With this sample size, the population must be tightly homogeneous to draw such conclusion ( eg only obese women, only underweight women etc.). Since this information is important and could inform future large sample size studies, the following major revisions would improve the quality of the manuscript.
 **1.** **The authors should try to make this manuscript more descriptive. Eg portraying histograms or bar charts with proportions of those with high BMI or BAI classifying them to have high body fat, who also have high body fat based on DEXA. Or correlation and regression figures for various BMI categories or any way just to make the manuscript more descriptive. This will help to suggest and not to conclude that BMI is likely superior to BAI pending future research with larger sample sizes or homogeneous populations. Would be good to have a manuscript that has looked at different angles of the 95 participants to convince that even though the sample size is small, it definitely suggest that BMI is more superior than BAI.**
Response: We have analysed the data further taking into account the reviewer comments. Our sample was first divided according to % fat levels (by DXA). Then using a using a clustered bar graph, the accuracy classifying adiposity using BMI categories versus BAI were shown. (Fig 1). Furthermore, ROC analysis was used to investigate which measure was better able to detect excess % BF (using a cut-off of 35%) (Fig 4). The results all revealed that BMI was more superior than BAI.

**Reviewer #2:** Comments on the paper entitled “Associations of body adiposity index, body mass index, waist circumference, and percentage of body fat in young female Emirati adults”

**1.Line 50, I suggest rewriting the sentence to read, Body Mass Index abbreviated as BMI, and not Body Mass Index also known as BMI**
Response: This has been revised and reads: “Body Mass Index abbreviated as BMI”.

**2.Line 150, How did you measure weight fluctuations**
Response: Weight fluctuations was gathered by asking participants, "Over the last 3 months, have you experienced any changes in your weight? If so, how many kilograms?". Further clarification has been added to the manuscript under the data collection section.

**3.Authors should mention the Names, Brands, Models, and Cities of manufacturers for all types of equipment and tools that were used in measuring all variables**
Response: This information has been added in the manuscript, material and methods section.

**4.It is stated that this study included healthy Emirati women attending “public universities” in the UAE who were chosen via a convenience sampling method (lines 123-124). It is also well explained that” The measurements were taken in the body composition laboratory at Zayed University in Dubai, UAE”. The clarifications are needed to disclose how participants from other universities were conveyed to the measurement center.**
Response: This was done via snowball sampling. It is now added to the manuscript.

**5.State if any statistical test for the normality of the data was carried out before running regression and correlation analysis**
Response: Normality was assessed using the using the Kolmogorov-Smirnov test- information has been added to the manuscript. % fat was normally distributed; however, WC, BAI and BMI were not. However, the distributions were approximately symmetric with one unusual point. As evidence of robustness, the removal of this point did not change the reported results. Therefore, this should not impact the statistical validity of the correlation and regression analyses.

**6.line 292 what is BIA?**
Response: This was a typing error and should have read BAI. It has been fixed.

**7.One of the aims of this study was to determine the relative accuracy of BAI when predicting %BF, However, this objective is not clearly shown through the subsequent sections of the manuscript, in other words the objective should be featured in data collection, analysis, and discussion.**
Response: Further analyses were carried out to test this aim, and was incorporated into the various sections of the manuscript. These include: (a) Paired sample t-tests for the sample overall as well as across various BMI categories and across different adiposity levels were used to test differences in % BF between DXA and BAI (Table 4), (b) Bland-Altman analysis was used to test individual agreement between BAI and % BF (DXA) (Figure 2) , (c) Linear regression analysis was carried out to test the best predictor of % fat (Table 5) (d) ROC analysis for sensitivity, specificity of BAI in detecting obesity by % BF (assessed by DXA) (Figure 4).
