# Supplementary material for: Associations of body adiposity index, body mass index, waist circumference, and percentage of body fat in young female Emirati adults
Source: PLoS One. 2024 May 6;19(5):e0302779. doi: 10.1371/journal.pone.0302779 (PMC11073684; doi:10.1371/journal.pone.0302779)
Supplement: Attachment — Submitted filename: Response to Reviewers.docx [file pone.0302779.s004.docx]

***Editor Comments***

Additional Editor Comments:

Upon further review, minor revisions have been recommended to enhance the manuscript's clarity and presentation. Specifically, it has been suggested that the current repetitive use of tables for presenting results could be diversified to improve readability and simplicity. Implementing these changes will further strengthen your manuscript.

Response: We thank the editor and reviewer for their comments, and have revised the presentation of results in two tables in the manuscript (1 table was converted into a figure and the other deleted) to enhance clarity and avoid repetitive use of tables. With these changes implemented, we hope that our paper is accepted for publication.
